# The association between triglyceride glucose-body mass index and overall survival in postoperative patient with lung cancer

**DOI:** 10.3389/fendo.2025.1528644

**Published:** 2025-07-16

**Authors:** Shanshan Cai, Hongquan Xing, Yihan Wang, Weichang Yang, Hongdan Luo, Xiaoqun Ye

**Affiliations:** Department of Respiratory and Critical Care Medicine, the Second Affiliated Hospital, Jiangxi Medical College, Nanchang University, Nanchang, Jiangxi, China

**Keywords:** lung cancer, TyG-BMI, prognostic biomarker, dynamic nomogram, overall survival

## Abstract

**Objective:**

Lung cancer continues to be one of the leading causes of cancer-related mortality, and the identification of effective prognostic markers is crucial for enhancing post-surgical outcomes. The present study was designed to investigate the association between the triglyceride-glucose body mass index (TyG-BMI) and postoperative overall survival (OS) rates in patients undergoing lung cancer surgery, while also evaluating its potential prognostic value for predicting postoperative outcomes.

**Methods:**

This study conducted a retrospective look at the data sourced from lung cancer patients undergone surgical procedures at the Second Affiliated Hospital of Nanchang University between 2016 and 2022. By dividing patients by TyG-BMI, the correlation between TyG-BMI and OS was determined via Cox regression modeling, Lasso regression, and Kaplan-Meier survival analyses. The link between TyG-BMI and OS regarding the dose-response was scrutinized by restricted cubic spline (RCS) analysis. A dynamic prognostic nomogram model based on TyG-BMI and other clinical factors was developed and validated.

**Results:**

The survival rates showed a significant variation between those with low and high TyG-BMI values, with the low TyG-BMI group having significantly better survival rates (P = 0.012). Multivariate analysis confirmed that smoking, pathological type, lymph node metastasis, N stage, and TyG-BMI were independent prognostic factors for OS. The nomogram model demonstrated robust predictive performance, achieving AUC values of 0.77, 0.81, and 0.86 for predicting OS at 24, 48, and 72 months, respectively, outperforming traditional TNM staging. Calibration and decision curve analyses further confirmed the model’s predictive accuracy and clinical utility.

**Conclusion:**

TyG-BMI is a valuable prognostic biomarker for assessing survival outcomes in lung cancer patients post-surgery. The predictive model based on TyG-BMI provides a valuable tool for the prognosis assessment of lung cancer. These findings need to be further validated, and the potential mechanism between TyG-BMI and lung cancer prognosis needs to be further investigated.

## 1 Introduction

Lung cancer ranks among the most common cancers globally, and its high incidence and mortality pose significant challenges to public health (1, 2). In 2025, Nearly 500 people will die from cancer every day, most of them from lung cancer, and it is estimated that lung cancer will accounts for approximately 124,730 deaths in the United States, continuing to be the leading cause of cancer mortality (3). Since early-stage lung cancer is usually asymptomatic, it is often diagnosed late, when metastasis has already occurred, contributing significantly to its high mortality. Surgery is the main treatment for early-stage lung cancer, but postoperative prognoses can vary widely. Research has shown that factors like tumor biology, laterality, and treatment methods affect postoperative prognosis (4), but the role of metabolic indicators remains unclear. Therefore, this study aims to identify reliable biomarkers to predict the prognosis of postoperative lung cancer patients, providing a more comprehensive understanding of the factors affecting their survival.

Therefore, finding out reliable biomarkers related to the prognosis of lung cancer is very important for clinical treatment and improving the survival rate of patients.

Insulin resistance (IR) refers to a pathological state where there is a decrease in insulin sensitivity among the body’s tissues or cells. This condition is frequently associated with the pathophysiological processes underlying metabolic disorders. Some individuals, even if not obese, are prone to impaired glucose tolerance and could be predisposed to IR (5–7). While the hyperinsulinemic-euglycemic clamp stands as the benchmark technique for measuring IR, this technique requires special equipment and skilled technicians, is costly and time-consuming, and involves multiple blood draws during the test, making it difficult for patients to accept. Currently, it is only used for scientific research and cannot be applied on a large scale in clinical settings. The triglyceride-glucose body mass index (TyG-BMI) derived from triglycerides (TG), fasting blood glucose (FBG), and body mass index (BMI), can offer a convenient and valuable alternative method for the hyperinsulinemic-euglycemic clamp in assessing IR (8).

TyG-BMI has long been recognized as a causal agent in the development of cardiovascular and metabolic diseases (9, 10). Some evidence now suggests that IR-related indicators have important predictive value for the prognosis of patients with malignancies (11, 12), especially the TyG-BMI index. As a combined index to evaluate IR status and muscle fat distribution of patients, it has been received increasing attention in cancer patients. Yin et al. (13) showed that IR substantially raises the likelihood of thyroid cancer, while Di Sebastiano et al. (14) revealed that hyperinsulinemia is tied to the emergence, growth, and malignancy of prostate cancer. Recently, some research has indicated that IR may as a condition that fosters the development of lung cancer. A cohort study focusing on male individuals revealed that those with IR exhibited a notably heightened risk of lung cancer following multivariate analysis. Similarly, a connection was found between the HOMA-IR index and lung cancer in a case-control analysis (15–17). Meanwhile, IR is also one of the most fundamental responses to injury and stress. In major surgeries such as colorectal surgery, up to 90% of insulin sensitivity may be lost postoperatively (18). Liu et al. (19) hypothesized that the TyG index is connected to long-term poor prognosis in postoperative patients.

Although the TyG-BMI index has been widely scientifically validated as a predictor of various diseases, given that the TyG-BMI can reflect an individual’s metabolic status and body composition characteristics, these features often have non-negligible importance in the prognosis assessment of cancer patients. Therefore, one might reasonably suggest that the TyG-BMI might also be a powerful tool for assessing the postoperative survival status of lung cancer patients. This hypothesis is not only given the TyG-BMI’s success in evaluating the prognosis of various other diseases but also stems from a deep understanding of the complexity of postoperative physiological and pathological changes in patients.

Existing studies on the TyG-BMI index and lung cancer have focused on its relationship with the onset of lung cancer, though its contribution to the postoperative prognosis of lung cancer patients remains unclear. To fill this research gap and provide more accurate and personalized prognosis prediction methods and treatment strategies for clinical practice, the purpose of this study is to deeply investigate the bond between the TyG-BMI index and post-surgical survival in lung cancer patients. Specifically, this study will focus for the first time on the potential role of preoperative TyG-BMI in the prognosis assessment of lung cancer surgery patients, by collecting and analyzing relevant clinical data to reveal how this key metabolic indicator affects the postoperative survival of lung cancer patients.

## 2 Patients and methods

### 2.1 Study subjects

**Figure 1:**
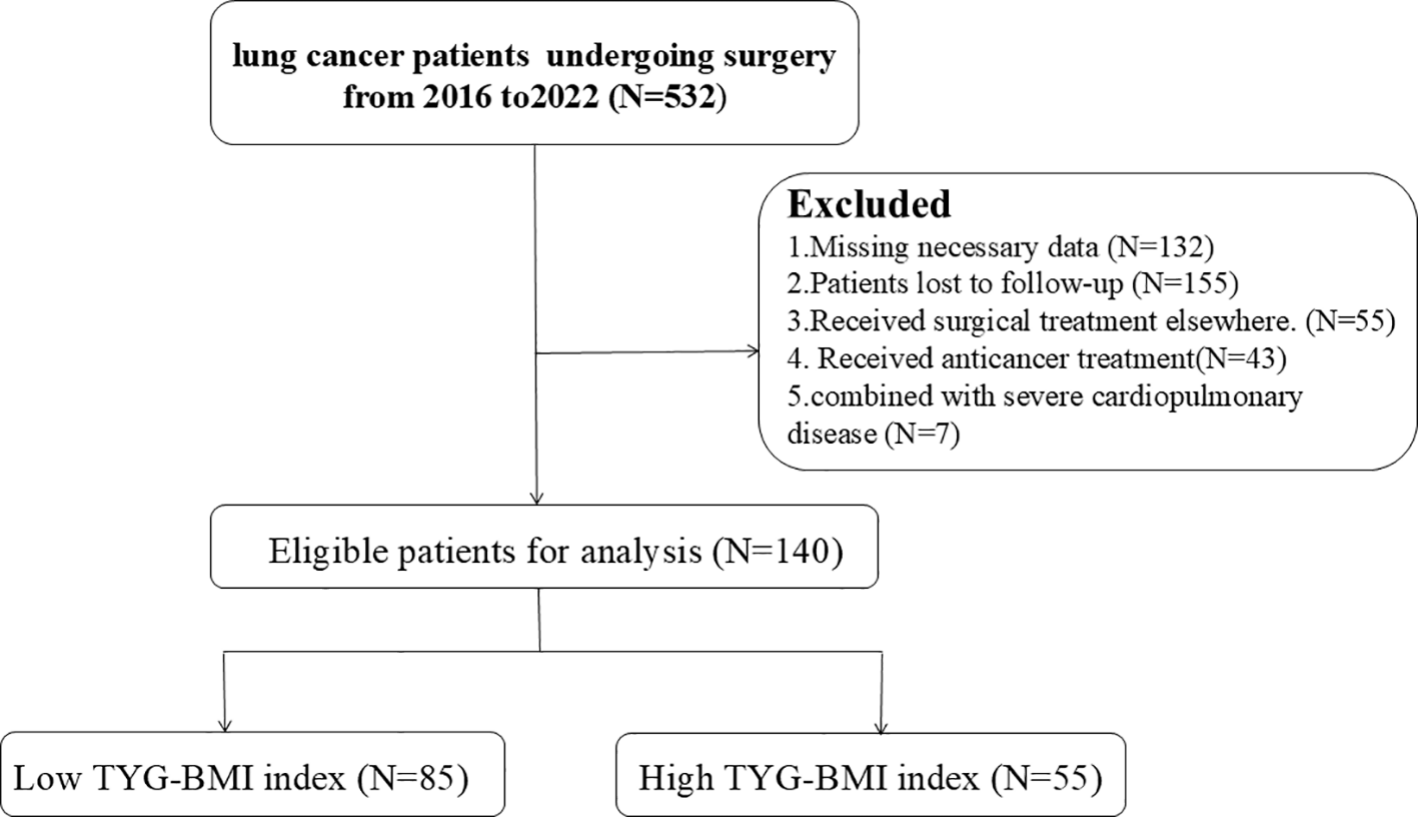
The flowchart of patient selection.

This observational analysis encompassed surgical lung cancer cases at the Second Affiliated Hospital of Nanchang University from 2016 to 2022. Inclusion criteria: (1) Pathologically or cytologically confirmed lung cancer diagnosis with complete medical records; (2) Underwent radical surgery with a smooth operation and no severe complications; (3) Aged over 18, with no preoperative serious complications like dysfunction of heart, liver, kidney, etc., and no history of other malignant tumors; (4) No history of long - term hormone use. Exclusions were based on: (1) Incomplete essential data, including TyG and BMI; (2) Lack of follow-up; (3) Surgery performed at other facilities; (4) Prior anticancer interventions; and (5) Concurrent severe cardiopulmonary conditions. **Figure 1** presents the methodology for patient selection.

The protocol was performed in accordance with the guidelines outlined in the Declaration of Helsinki and was approved by the Ethics Committee of The Second Affiliated Hospital of Nanchang University. Since the study was a retrospective study, most of the study subjects have died or lost contacts, and all statistics were anonymous, so the Ethics Committee of The Second Affiliated Hospital of Nanchang University agreed to waive the need for informed consent.

### 2.2 Data collection

Initial data potentially impacting lung cancer outcomes were gathered, comprising: (1) demographic details: age and gender; (2) medical background: smoking and drinking habits, as well as history of cardiovascular disease and diabetes; (3) pathological information: primary tumor location, tumor dimensions, volume, histological type, Ki67 levels, tumor invasion, and TNM staging; (4) laboratory results: The TyG index was generated according to the formula ln [TG (mg/dL) × FBG (mg/dL)]/2, and the TyG-BMI score was constructed by adding BMI to the TyG index. These measurements were based on initial post-admission assessments before any treatment was initiated.

### 2.3 Assessment of TyG-BMI

Collect patients’ height and weight at admission to calculate BMI, and obtain their preoperative triglyceride and fasting blood glucose levels. Through the application of K-means clustering algorithm, participants were categorized into two distinct groups based on their TyG-BMI levels: one with elevated TyG-BMI values and another with lower TyG-BMI values. A detailed comparative analysis was performed to examine the baseline characteristics of these two cohorts. To evaluate the correlation between TyG-BMI and OS, both Cox proportional hazards regression and Lasso regression models were utilized. In addition, subgroup analyses were conducted to delve deeper into the relationship between TyG-BMI and OS across various demographic and clinical contexts. Furthermore, the non-linear relationship between TyG-BMI, treated as a continuous variable, and OS was explored using restricted cubic spline (RCS) analysis.

### 2.4 Statistical methods

The statistical analysis was conducted using SPSS software, version 25.0. For categorical data, the results were presented as counts and percentages, and group differences were evaluated using either Pearson’s Chi-square test or Fisher’s exact test, depending on the data distribution. Continuous variables that were normally distributed were expressed as the mean with standard deviation, and group comparisons were made using the t-test. In cases where the continuous variables were not normally distributed, the median and interquartile range (IQR) were reported, and the Wilcoxon rank-sum test was applied to determine the statistical differences. The significance level was defined as a P < 0.05.

### 2.5 Establishment and evaluation of a prognostic model

A predictive model for lung cancer patient survival was formulated based on the outcomes of Lasso regression analysis. The model’s prognostic reliability was appraised with receiver operating characteristic (ROC) curves and the area under the curve (AUC) as a measure. Calibration diagrams were constructed to verify the consistency between forecasted and observed survival data. decision curve analysis (DCA) and clinical impact curve (CIC) were performed to determine the model’s clinical utility. For visualizing the prognostic nomogram, an interactive web application was created using Shiny (version 1.7.2), which can be accessed at https://cai123.shinyapps.io/dynnomapp/).

## 3 Results

### 3.1 Baseline characteristics

Enrollment in the study resulted in 140 individuals, comprising 110 males and 30 females, with an average age of 61 ± 10 years. Participants were followed up for a median duration of 36 months. **Table 1** displays the demographic and clinical features of the study’s participants. There were significant statistical differences between the two groups in gender, pathological type, and lymph node involvement (p < 0.05). No notable differences were identified for the remaining variables.

**Table 1:** Patient demographics and baseline characteristics of TYG-BMI groups.

Characteristic	TYG-BMI_group			p-value
	Overall, N = 140^1^	Low group, N = 85^1^	High group, N = 55^1^	
Age. Years	61 ± 10	61 ± 9	61 ± 11	0.705^2^
Gender				0.028^3^
Male	110 (78.6%)	72 (84.7%)	38 (69.1%)	
Female	30 (21.4%)	13 (15.3%)	17 (30.9%)	
Smoking				0.466^3^
Yes	69 (49.3%)	44 (51.8%)	25 (45.5%)	
No	71 (50.7%)	41 (48.2%)	30 (54.5%)	
Drinking				0.552^3^
Yes	29 (20.7%)	19 (22.4%)	10 (18.2%)	
No	111 (79.3%)	66 (77.6%)	45 (81.8%)	
Cardiovascular disease				0.396^3^
Yes	36 (25.7%)	24 (28.2%)	12 (21.8%)	
No	104 (74.3%)	61 (71.8%)	43 (78.2%)	
Diabetes				0.158^3^
Yes	20 (14.3%)	15 (17.6%)	5 (9.1%)	
No	120 (85.7%)	70 (82.4%)	50 (90.9%)	
Pathological Type				0.033^4^
LUSC	85 (60.7%)	49 (57.6%)	36 (65.5%)	
LUAD	46 (32.9%)	27 (31.8%)	19 (34.5%)	
Others	9 (6.4%)	9 (10.6%)	0 (0.0%)	
Primary Site				0.969^4^
Main bronchus	9 (6.4%)	6 (7.1%)	3 (5.5%)	
Upper lobe	68 (48.6%)	42 (49.4%)	26 (47.3%)	
Lower lobe	44 (31.4%)	26 (30.6%)	18 (32.7%)	
Others	19 (13.6%)	11 (12.9%)	8 (14.5%)	
Laterality				0.109^3^
Left	58 (41.4%)	41 (48.2%)	17 (30.9%)	
Right	67 (47.9%)	35 (41.2%)	32 (58.2%)	
Others	15 (10.7%)	9 (10.6%)	6 (10.9%)	
Tumor Length (cm)	3.43 (2.10, 5.12)	3.15 (2.10, 4.50)	3.80 (2.20, 5.50)	0.255^5^
Volume (cm3)	13 (3, 43)	12 (3, 43)	14 (2, 46)	0.875^5^
Ki67	30 (15, 60)	30 (10, 60)	40 (25, 60)	0.185^5^
Bronchus Invasion				0.694^3^
Yes	33 (23.6%)	21 (24.7%)	12 (21.8%)	
No	107 (76.4%)	64 (75.3%)	43 (78.2%)	
Vascular Invasion				0.764^3^
No	105 (75.0%)	63 (74.1%)	42 (76.4%)	
Yes	35 (25.0%)	22 (25.9%)	13 (23.6%)	
Nerve invasion				>0.999^4^
Yes	10 (7.1%)	6 (7.1%)	4 (7.3%)	
No	130 (92.9%)	79 (92.9%)	51 (92.7%)	
Pleura Invasion				0.787^3^
Yes	19 (13.6%)	11 (12.9%)	8 (14.5%)	
No	121 (86.4%)	74 (87.1%)	47 (85.5%)	
Lmphatic Metastasis				0.042^3^
Yes	90 (64.3%)	49 (57.6%)	41 (74.5%)	
No	50 (35.7%)	36 (42.4%)	14 (25.5%)	
T stage				0.102^3^
T1	39 (27.9%)	27 (31.8%)	12 (21.8%)	
T4	31 (22.1%)	18 (21.2%)	13 (23.6%)	
T3	21 (15.0%)	8 (9.4%)	13 (23.6%)	
T2	49 (35.0%)	32 (37.6%)	17 (30.9%)	
N stage				0.105^3^
N0	50 (35.7%)	36 (42.4%)	14 (25.5%)	
N1	22 (15.7%)	13 (15.3%)	9 (16.4%)	
N2	48 (34.3%)	23 (27.1%)	25 (45.5%)	
N3	20 (14.3%)	13 (15.3%)	7 (12.7%)	
M stage				0.904^3^
M0	119 (85.0%)	72 (84.7%)	47 (85.5%)	
M1	21 (15.0%)	13 (15.3%)	8 (14.5%)	

^1^Mean ± SD; n (%); Median (IQR).

^2^Welch Two Sample t-test.

^3^Pearson’s Chi-squared test.

^4^Fisher’s exact test.

^5^Wilcoxon rank sum test.

### 3.2 Prognostic value of TyG-BMI index in lung cancer

**Figure 2:**
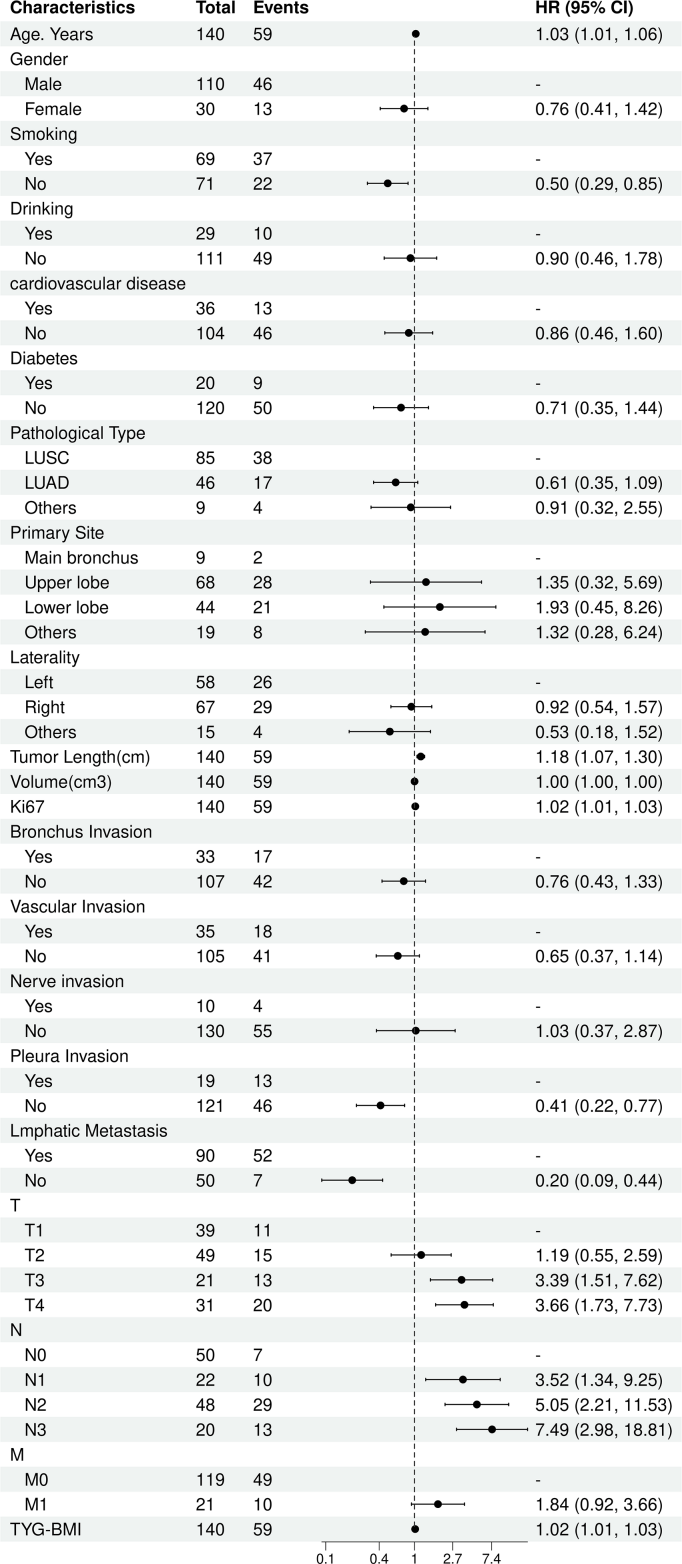
Univariate COX regression analysis for overall survival in lung cancer.

**Figure 3:**
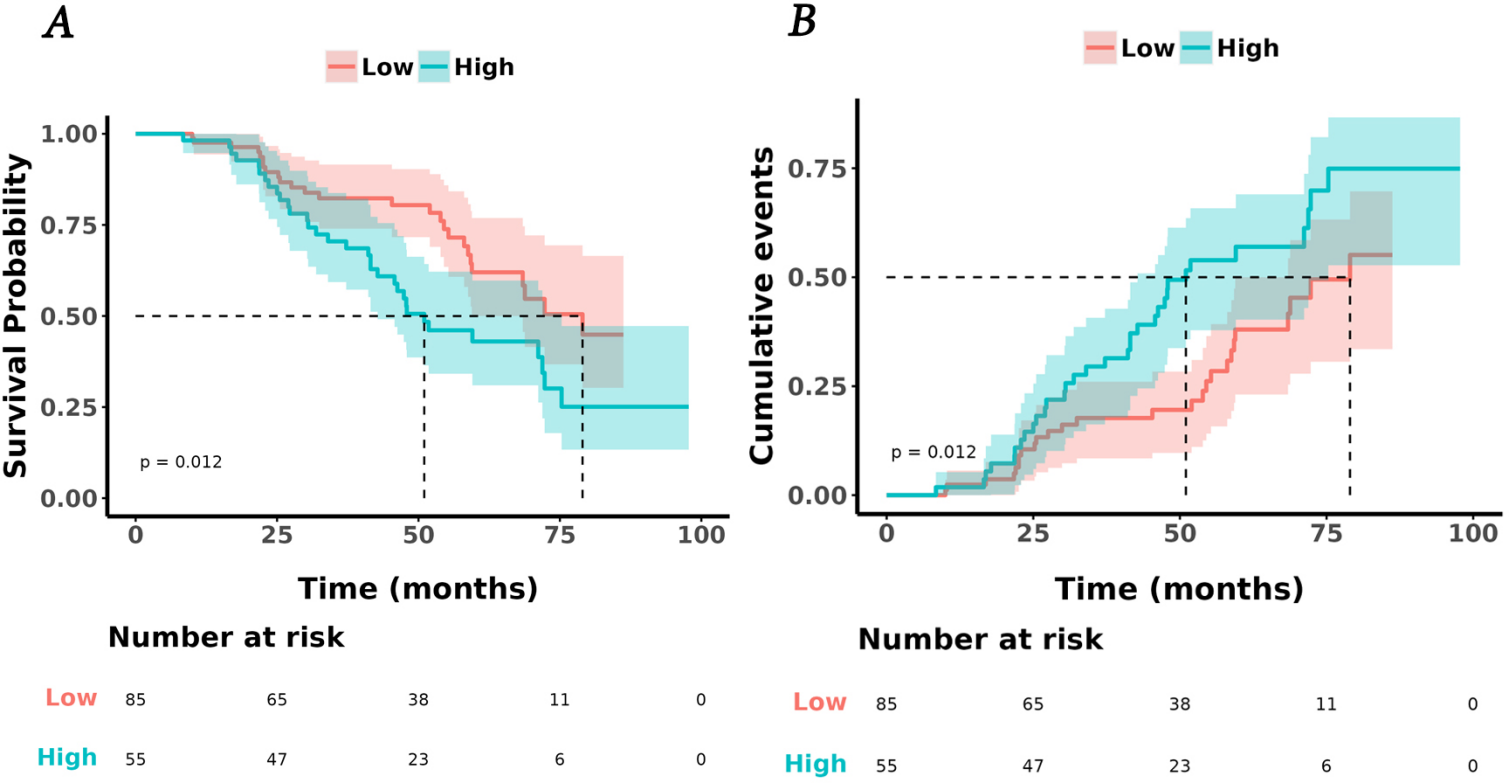
Kaplan-Meier analysis of TyG and lung cancer. **(A)** survival curves for overall survival in high and low TyG-BMI index groups. **(B)** cumulative events for death in high and low TyG-BMI index groups.

The univariate Cox analysis confirmed that TyG-BMI was a prognostic factor for OS (HR = 1.02, P = 0.002). Additionally, age, smoking, tumor length, tumor volume, Ki67 expression, lmphatic metastasis, T and N stage were also prognostic factors for OS (**Figure 2**, **Table 2**). The KM curve highlighted that subjects with high TyG-BMI index faced a considerably reduced OS compared to subjects with low TyG-BMI index (P = 0.012) (**Figure 3**). The 24-, 48-, and 72-month OS rates were found to be diminished in the high TyG-BMI group in comparison with the low TyG-BMI group (85.5% vs. 89.5%, 50.6% vs. 80.4%, 34.4% vs. 54.7%) (**Table 3**).

**Table 2:** Univariate analysis of influencing factors (Cox regression).

Characteristic	N	Event N	HR^1^	95% CI^1^	p-value
Age. Years	140	59	1.03	1.01, 1.06	0.020
Gender					
Male	110	46	—	—	
Female	30	13	0.76	0.41, 1.42	0.392
Smoking					
No	71	22	—	—	
Yes	69	37	2.00	1.18, 3.40	0.010
Drinking					
No	111	49	—	—	
Yes	29	10	1.11	0.56, 2.20	0.763
Cardiovascular disease					
No	104	46	—	—	
Yes	36	13	1.16	0.62, 2.16	0.638
Diabetes					
No	120	50	—	—	
Yes	20	9	1.42	0.70, 2.89	0.336
Pathological Type					
LUSC	85	38	—	—	
LUAD	46	17	0.61	0.35, 1.09	0.095
Others	9	4	0.91	0.32, 2.55	0.856
Primary Site					
Main bronchus	9	2	—	—	
Upper lobe	68	28	1.35	0.32, 5.69	0.684
Lower lobe	44	21	1.93	0.45, 8.26	0.374
Others	19	8	1.32	0.28, 6.24	0.727
Laterality					
Left	58	26	—	—	
Right	67	29	0.92	0.54, 1.57	0.762
Others	15	4	0.53	0.18, 1.52	0.235
Tumor Length(cm)	140	59	1.18	1.07, 1.30	0.023
Volume(cm3)	140	59	1.00	1.00, 1.00	0.048
Ki67	140	59	1.02	1.01, 1.03	0.008
Bronchus Invasion					
No	107	42	—	—	
Yes	33	17	1.32	0.75, 2.32	0.333
Vascular Invasion					
No	105	41	—	—	
Yes	35	18	1.54	0.88, 2.70	0.130
Nerve invasion					
No	130	55	—	—	
Yes	10	4	0.97	0.35, 2.68	0.948
Pleura Invasion					
No	121	46	—	—	
Yes	19	13	2.41	1.29, 4.51	0.006
Lmphatic Metastasis					
No	50	7	—	—	
Yes	90	52	5.03	2.28, 11.09	0.007
T					
T1	39	11	—	—	
T2	49	15	1.19	0.55, 2.59	0.663
T3	21	13	3.39	1.51, 7.62	0.003
T4	31	20	3.66	1.73, 7.73	0.006
N					
N0	50	7	—	—	
N1	22	10	3.52	1.34, 9.25	0.011
N2	48	29	5.05	2.21, 11.53	0.003
N3	20	13	7.49	2.98, 18.81	0.002
M					
M0	119	49	—	—	
M1	21	10	1.84	0.92, 3.66	0.083
TYG-BMI	140	59	1.02	1.01, 1.03	0.002

^1^HR, Hazard Ratio; CI, Confidence Interval.

**Table 3:** Kaplan-meier estimates for survival rates (95% CI).

Characteristic	N	Event N	24-month	48-month	72-month	p-value^1^
Overall	140	59	87.8% (82.4%, 93.6%)	66.6% (58.6%, 75.8%)	45.1% (35.2%, 57.8%)	
TYG-BMI group	140	59				0.012
Low			89.5% (82.8%, 96.7%)	80.4% (71.6%, 90.3%)	54.7% (41.5%, 72.1%)	
High			85.5% (76.6%, 95.3%)	50.6% (38.6%, 66.3%)	34.4% (21.9%, 54.0%)	

^1^Log-rank test.

**Figure 4:**
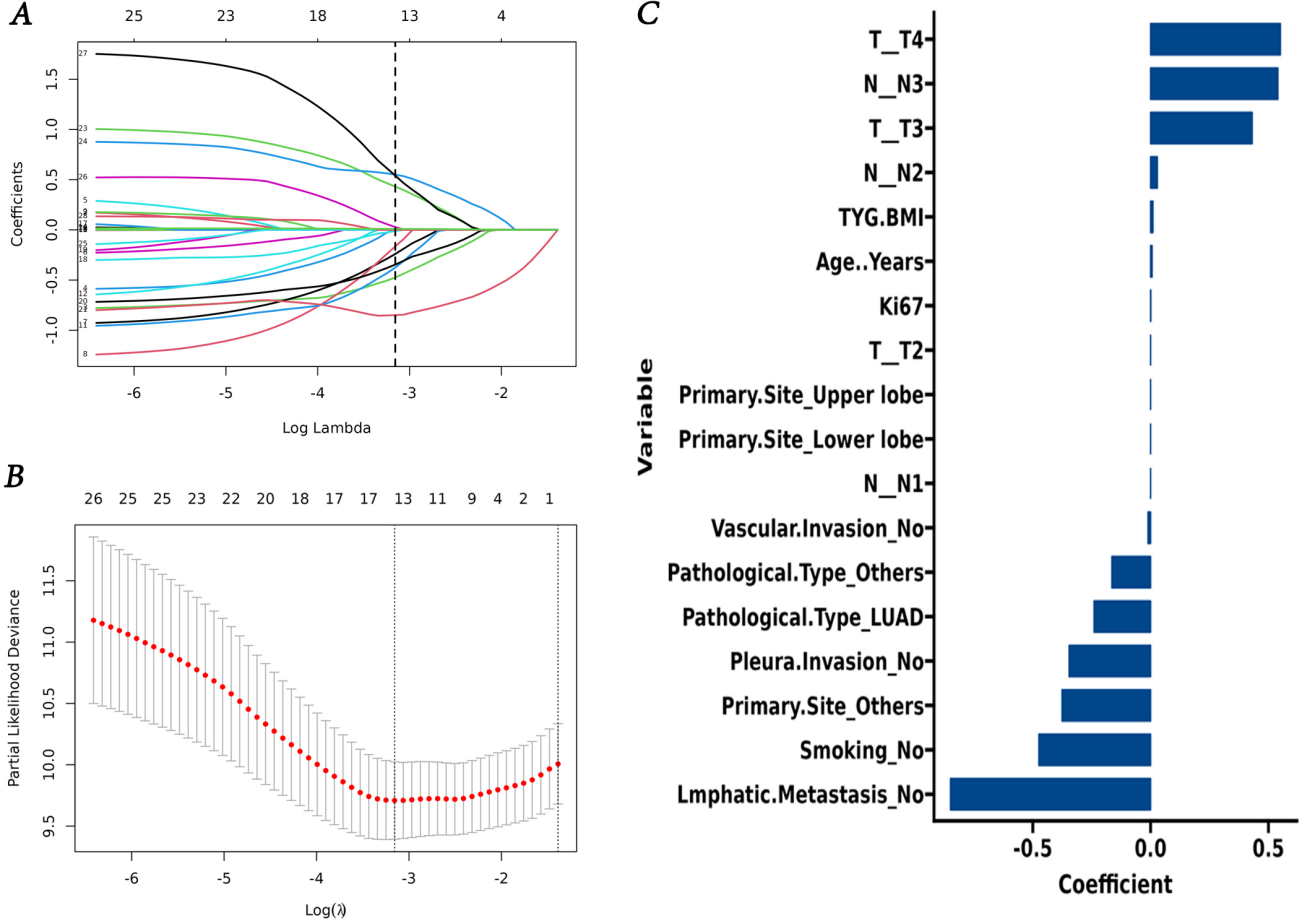
Screening of variables based on Lasso regression. **(A)** The variation characteristics of the coefficient of variables; **(B)** the selection process of the optimum value of the parameter λ in the Lasso regression model by cross-validation method. **(C)** Lasso regression coefficient of variable.

Lasso regression analysis indicated that age, smoking, Ki67 expression, T and N stage, primary site, pathological type, lmphatic metastasis, pleural invasion, vascular invasion, and TyG-BMI were all prognostic factors for OS (**Figure 4**). Multivariate COX analysis of these variables further identified smoking, pathological type, lmphatic metastasis, N stage, and TyG-BMI as independent predictors of OS (**Table 4**).

**Table 4:** Cox regression with variable reduction (variables selected by regularized regression).

Characteristic	N	Event N	HR^1^	95% CI^1^	p-value
Age. Years	140	59	1.00	0.97, 1.04	0.961
Smoking					
Yes	69	37	—	—	
No	71	22	0.39	0.20, 0.76	0.005
Pathological Type					
LUSC	85	38	—	—	
LUAD	46	17	0.42	0.21, 0.86	0.016
Others	9	4	0.37	0.11, 1.24	0.106
Primary Site					
Main bronchus	9	2	—	—	
Upper lobe	68	28	0.69	0.15, 3.28	0.642
Lower lobe	44	21	0.63	0.12, 3.23	0.580
Others	19	8	0.22	0.04, 1.28	0.092
Ki67	140	59	1.00	0.98, 1.01	0.665
Vascular Invasion					
Yes	35	18	—	—	
No	105	41	0.82	0.43, 1.56	0.541
Pleura Invasion					
Yes	19	13	—	—	
No	121	46	0.49	0.23, 1.06	0.070
Lmphatic Metastasis					
Yes	90	52	—	—	
No	50	7	0.09	0.03, 0.29	0.003
T					
T1	39	11	—	—	
T2	49	15	1.00	0.43, 2.35	0.995
T3	21	13	2.24	0.89, 5.66	0.088
T4	31	20	2.14	0.88, 5.20	0.095
N					
N0	50	7	—	—	
N1	22	10	1.24	0.08, 0.72	0.011
N2	48	29	1.33	0.13, 0.83	0.019
N3	20	13			
TYG-BMI	140	59	1.01	1.00, 1.03	0.026

^1^HR, Hazard Ratio; CI, Confidence Interval.

**Figure 5:**
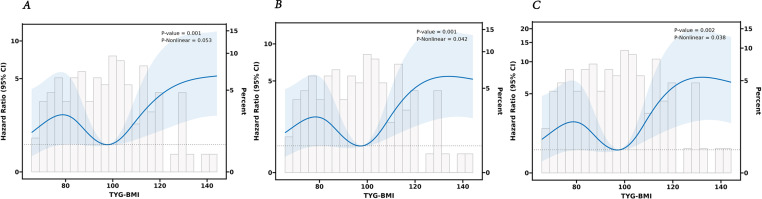
The RCS analysis between the TyG-BMI index and overall survival of lung cancer. **(A)** unadjusted. **(B)** adjusted for Age, Years, Gender. **(C)** adjusted for Age, Years, Gender, Smoking, Primary Site, Laterality, Ki67, Bronchus Invasion, Vascular Invasion, Pleura Invasion, Volume, Nerve invasion, and Pathological Type.

Furthermore, we established three Cox proportional hazards models. The findings indicated that TyG-BMI is a prognostic factor for lung cancer OS. Upon adjusting for age, gender, and other variables potentially impacting lung cancer outcomes, TyG-BMI continued to be acknowledged as a prognostic factor (**Table 5**). Employing the RCS Cox model, we delved deeper into the potential linear link between TyG-BMI and lung cancer outcomes. TyG-BMI showed a non-linear association with OS in both the model 2 (P = 0.001, P-nonlinear = 0.042) and the model 3 (P = 0.002, P-nonlinear = 0.038) (**Figure 5**).

**Table 5:** Cox proportional hazard models for overall survival.

Characteristic	N	Event N	HR^1^	95% CI1	p-value
TYG-BMI_group (unadjusted)					
Low	85	26	—	—	
High	55	33	1.93	1.15, 3.23	0.012
TYG-BMI_group (model 1)*					
Low	85	26	—	—	
High	55	33	2.09	1.24, 3.53	0.005
TYG-BMI_group (model 2)*					
Low	85	26	—	—	
High	55	33	1.97	1.11, 3.51	0.020

^1^HR, Hazard Ratio; CI, Confidence Interval.

* Model 1 adjusted for Age. Years, Gender.

* Model 2 adjusted for Age. Years, Gender, Smoking, Primary Site, Laterality, Ki67, Bronchus Invasion, Vascular Invasion, Pleura Invasion, Volume(cm3), Nerve invasion, and Pathological Type.

**Figure 6:**
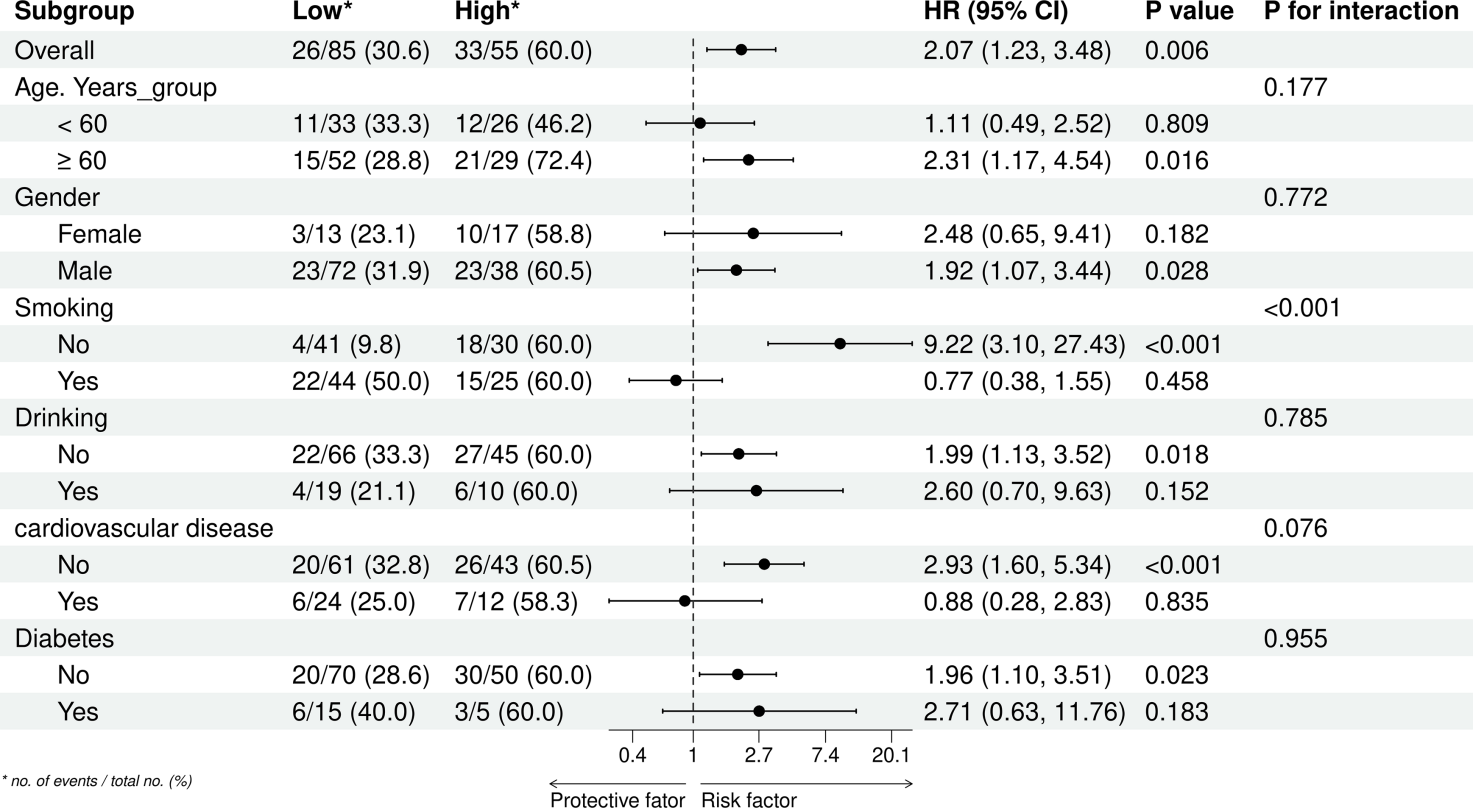
Subgroup analyses of the association between the TyG-BMI index and overall survival of lung cancer. *no. of events/total no. (%).

To provide a more comprehensive assessment of the relationship between TyG-BMI and lung cancer prognosis, we conducted subgroup and interaction analyses based on age, gender, smoking, drinking, cardiovascular disease, and diabetes group. The results (**Figure 6**, **Supplementary Table S1**) showed a significant interaction effect only within the smoking subgroup (P for interaction < 0.001). In contrast, no interactions were recorded in the other subgroups (P for interaction > 0.05).

### 3.3 Nomogram model of lung cancer prognosis

**Figure 7:**
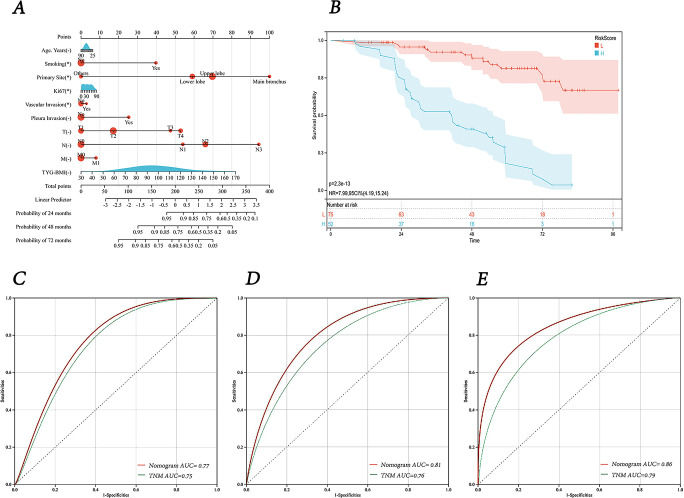
Nomogram and its receiver operating characteristic curves. **(A)** Nomogram for predicting 24-, 48-, and 72-months survival. **(B)** Kaplan-Meier curves for OS in lung cancer patients stratified by risk category. **(C-E)** Receiver operating characteristic curves and area under the curve (AUC) for predicting 24-, 48-, and 72-months survival of the nomogram model compared with TNM staging.

Using the results from the Lasso regression analysis, we created a nomogram to forecast patient prognosis (**Figure 7A**). The nomogram includes age, smoking, Ki67 expression, TNM stage, primary site, pleural invasion, vascular invasion, and TyG-BMI (pathological type and lmphatic metastasis were excluded as they are covered under TNM stage). As shown by the Kaplan-Meier survival analysis, patients designated high-risk showed a considerably poorer OS than those designated low-risk (P < 0.0001) (**Figure 7B**). The AUC of model for 24-, 48-, and 72-month OS were 0.77, 0.81, and 0.86, respectively, significantly outperforming the TNM staging system, which had AUCs of 0.75, 0.76, and 0.79 (**Figures 7C–E**). Finally, a user-friendly online application of this model for assessing lung cancer prognosis has been developed and is freely accessible via a cloud platform (https://cai123.shinyapps.io/dynnomapp/).

**Figure 8:**
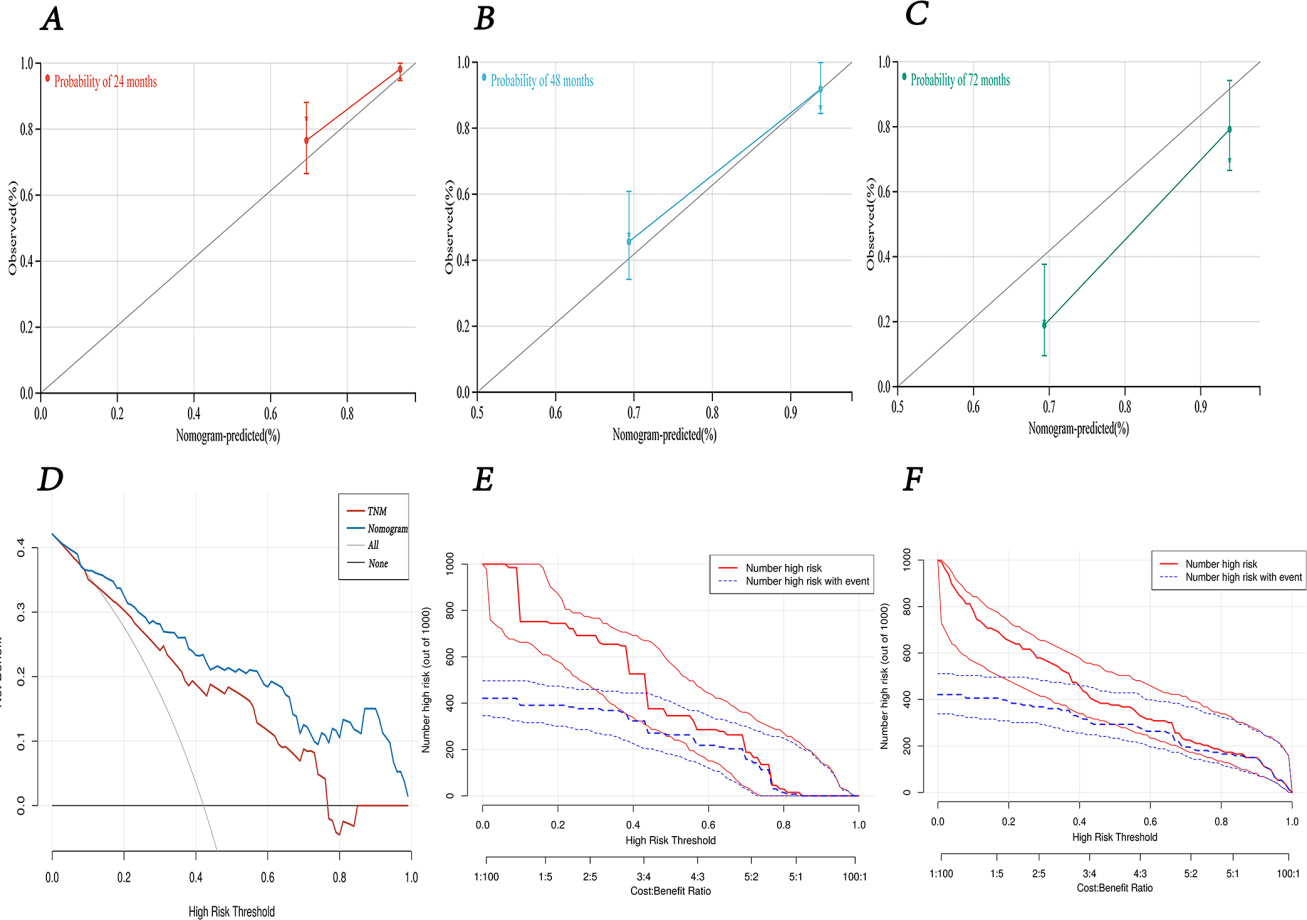
The predictive performance of nomogram model. **(A-C)** calibration curves for 24-, 48-, and 72-months survival. **(D)** decision curve analysis for overall survival of the nomogram model compared with TNM staging. **(E)** clinical impact curve of TNM staging. **(F)** clinical impact curve of nomogram model.

The calibration curves at 24, 48, and 72 months demonstrated a high degree of consistency between the nomogram’s predictions and the actual observed outcomes (**Figures 8A–C**). The DCA and CIC results indicated that our created nomogram prediction model outperformed the TNM staging system, providing superior clinical benefits and demonstrating reasonable practicality for clinical application (**Figures 8D–F**).

## 4 Discussion

Lung cancer ranks as one of the most common malignant tumors globally and is a primary contributor to cancer-related deaths (20). Therefore, accurate prognostic assessment of lung cancer is critical for improving patient outcomes. Currently, the TNM staging system is the most commonly used tool for tumor evaluation. However, with the advancement of research, it has become evident that lung cancer prognosis is determined not solely by the anatomical extent of the disease but also by a multitude of clinical, biological and genetic factors (21, 22). Thus, it is essential to integrate these new key factors to develop methods that can more accurately assess the prognosis of lung cancer patients. Although existing studies have highlighted the potential value of molecular genetic markers in prognostic evaluation, these methods are often expensive and challenging to implement routinely in clinical practice (23, 24). Therefore, it is crucial need to develop simple, practical, and effective prognostic biomarkers for lung cancer.

The TyG-BMI, calculated based on TG, FPG, and BMI, has emerged as a robust and convenient tool for assessing IR. Many studies have illustrated a pronounced tie between TyG-BMI and the occurrence and advancement of multiple types of cancer, including lung cance (25–28). Yan et al. (29) found that the TyG index in lung cancer patients was notably elevated compared to the control cohort (8.42 vs. 8.00, P < 0.01). Furthermore, the TyG index was identified as a predictor of lung cancer susceptibility, with an OR of 3.651 (P < 0.001). Similarly, Wang et al. (30) observed that lung cancer patients had a higher average TyG-BMI index than the control group (201 vs. 174, P < 0.01). Additionally, the incidence of lung cancer was markedly higher in the high TyG-BMI group as opposed to the medium and low TyG-BMI groups, with rates of 60.46%, 12.61%, and 26.83% respectively (P < 0.01). In contrast, a study leveraging data from the UK Biobank did not reveal a significant link between the TyG index and lung cancer risk (31). These conflicting results may stem from differences in lifestyle, dietary habits, geographical factors, and genetics across different ethnic groups. Nevertheless, these studies suggest a potential link between TyG-BMI and the occurrence of lung cancer, while the relationship between TyG-BMI and lung cancer prognosis remains unclear.

Our investigation is groundbreaking in examining the tie between TyG-BMI and survival prognosis in lung cancer patients who have undergone surgery. With Cox regression and Lasso analysis, we established a pronounced link between TyG-BMI and survival. KM curve analysis further substantiated that patients with elevated TyG-BMI experienced a notably decreased survival time compared to those with lower TyG-BMI. These insights suggest that TyG-BMI could potentially act as a beneficial indicator for the prognosis assessment of lung cancer patients, aiding clinicians in developing more appropriate treatment strategies and prognostic evaluations. Additionally, our analysis using RCS Cox proportional hazards regression model revealed a positive nonlinear relationship between the TyG-BMI and OS, suggesting that clinical practice should place greater emphasis on monitoring the trends in TyG-BMI values.

To facilitate the clinical application of TyG-BMI in the prognostic evaluation of lung cancer, we crafted a prognostic nomogram model and evaluated its predictive accuracy drawing upon the outcomes of Lasso regression analysis. The model exhibited AUC values of 0.77, 0.81, and 0.86 for predicting OS at 24, 48, and 72 months, respectively, which are significantly higher than those of the TNM staging system. Furthermore, calibration and decision curve analyses demonstrated that the model possesses strong predictive accuracy and clinical utility. These findings provide a novel and more precise method for assessing lung cancer prognosis, contributing valuable insights for guiding clinical practice.

Although the specific mechanisms by which TyG-BMI affects lung cancer are not yet fully understood, existing research has identified several potential mechanisms related to IR. IR can lead to a decrease in insulin bioactivity, which subsequently triggers an increase in insulin secretion to compensate for its declining function. Numerous studies have indicated that the levels of IGF-1 and IGF-2 are increased in the tumor tissues of lung cancer, and these growth factors promote cell growth, proliferation, invasion, and apoptosis suppression by activating multiple signaling pathway, including PI3K/Akt and Ras/MAPK (32). Additionally, IR may enhance inflammatory responses in tumor tissues and increase the secretion of inflammatory factors, which further promote tumor growth, invasion, and metastasis (33). Furthermore, IR could also trigger an overproduction of reactive oxygen species (ROS), which can cause oxidative damage to DNA and somatic mutations, thereby increasing the risk of tumor development (34). Insulin resistance not only significantly impacts the biological characteristics of tumor cells but also has a substantial effect on the tumor microenvironment, facilitating the progression of lung cancer (35). Together, these mechanisms highlight insulin resistance’s involvement in the intensification of lung cancer.

The study demonstrates the pivotal position of TyG-BMI in the evaluation of lung cancer prognosis and presents a dynamic prognostic nomogram model for lung cancer. However, this study does have some limitations, the sample may lack diversity, as it primarily comes from specific regions or populations, which restricts the generalizability of the findings. The sample size might be too small, resulting in insufficient statistical power to detect potential associations or effects. Retrospective studies rely on past medical records, which can be incomplete or inaccurate. The absence of long-term follow-up data makes it hard to evaluate the sustained impact of TYG-BMI on survival. Additionally, other factors affecting overall survival, such as lifestyle, treatment plans, and comorbidities, may not be fully considered. These factors could interact with TYG-BMI, affecting the accuracy of the results. To further confirm our findings, future research should endeavor to conduct larger, multi-center, prospective studies and explore additional factors that may influence lung cancer prognosis. Additionally, a deeper investigation into the specific mechanisms linking the TyG-BMI to lung cancer prognosis will provide stronger theoretical support pertaining to the preclusion and medical management of lung cancer.

The TyG-BMI index has demonstrated its utility as a significant predictor for preoperative lung cancer prognosis. Our research suggests that individuals with elevated TyG-BMI values are associated with significantly worse postoperative survival rates. Notably, this association underscores the importance of integrating metabolic indicators into prognostic assessments. Additionally, the TyG-BMI index provides clinicians with a new dimension for customizing treatment strategies, highlighting the need for a comprehensive approach that includes monitoring triglyceride and glucose levels. This study advocates for further exploration of the role of TyG-BMI in lung cancer prognosis to enhance patient management and outcomes.

## 5 Conclusion

In conclusion, our study emphasizes the prognostic significance of the TyG-BMI index for postoperative lung cancer patients. We thought that TyG-BMI index can provide valuable insights into patient outcomes, serving as a useful supplement to traditional clinical and pathological factors. Nevertheless, our study also recognizes certain limitations, such as sample size and study design, and calls for further research to validate the findings and investigate additional prognostic factors. Overall, incorporating metabolic markers like TYG-BMI index into clinical practice could enhance personalized treatment strategies and potentially improve survival rates for lung cancer patients.

## Data Availability

The raw data supporting the conclusions of this article will be made available by the authors, without undue reservation.
