# Supplementary material for: The association between triglyceride glucose-body mass index and overall survival in postoperative patient with lung cancer
Source: Front Endocrinol (Lausanne). 2025 Jul 16;16:1528644. doi: 10.3389/fendo.2025.1528644 (PMC12307163; doi:10.3389/fendo.2025.1528644)
Supplement: Supplementary file 1 [file Table1.docx]

Table S1:Subgroup Analysis

| Subgroup | Low^1^ | High^1^ | Crude HR (95% CI) | P value | P for interaction |
| --- | --- | --- | --- | --- | --- |
| Overall | 26/85 (30.6) | 33/55 (60.0) | 1.93 (1.15-3.23) | 0.012 |  |
| Age. Years_group |  |  |  |  | 0.256 |
| < 60 | 11/33 (33.3) | 12/26 (46.2) | 1.34 (0.59-3.05) | 0.48 |  |
| ≥ 60 | 15/52 (28.8) | 21/29 (72.4) | 2.56 (1.32-4.98) | 0.006 |  |
| Gender |  |  |  |  | 0.411 |
| Female | 3/13 (23.1) | 10/17 (58.8) | 3.06 (0.84-11.16) | 0.091 |  |
| Male | 23/72 (31.9) | 23/38 (60.5) | 1.80 (1.01-3.22) | 0.046 |  |
| Smoking |  |  |  |  | 0.004 |
| No | 4/41 (9.8) | 18/30 (60.0) | 6.70 (2.27-19.81) | 0.001 |  |
| Yes | 22/44 (50.0) | 15/25 (60.0) | 1.03 (0.53-2.00) | 0.921 |  |
| Drinking |  |  |  |  | 0.571 |
| No | 22/66 (33.3) | 27/45 (60.0) | 1.82 (1.03-3.19) | 0.038 |  |
| Yes | 4/19 (21.1) | 6/10 (60.0) | 2.80 (0.76-10.27) | 0.121 |  |
| cardiovascular disease |  |  |  |  | 0.45 |
| No | 20/61 (32.8) | 26/43 (60.5) | 1.77 (0.99-3.18) | 0.056 |  |
| Yes | 6/24 (25.0) | 7/12 (58.3) | 2.96 (0.97-9.04) | 0.057 |  |
| Diabetes |  |  |  |  | 0.502 |
| No | 20/70 (28.6) | 30/50 (60.0) | 2.15 (1.22-3.79) | 0.008 |  |
| Yes | 6/15 (40.0) | 3/5 (60.0) | 1.57 (0.39-6.37) | 0.525 |  |
| ^1^no. of events / total no. (%) | | | | | |
